# Factors Associated with Diet Quality among Adolescents in a Post-Disaster Area: A Cross-Sectional Study in Indonesia

**DOI:** 10.3390/nu15051101

**Published:** 2023-02-22

**Authors:** Nikmah Utami Dewi, Ali Khomsan, Cesilia Meti Dwiriani, Hadi Riyadi, Ikeu Ekayanti, Diah Ayu Hartini, Rasyika Nurul Fadjriyah

**Affiliations:** 1 Department of Community Nutrition, Faculty of Human Ecology, IPB University, Bogor 16680, Indonesia; 2 Department of Nutrition, Faculty of Public Health, University of Tadulako, Palu 94148, Indonesia; 3 Department of Nutrition, Health Polytechnic of Palu, Palu 94148, Indonesia; 4 Department of Public Health, Faculty of Public Health, University of Tadulako, Palu 94148, Indonesia

**Keywords:** adolescent, diet quality, food security, post-disaster, vulnerable group

## Abstract

The diet quality of adolescents in low-middle-income countries is low. Especially in post-disaster areas, adolescents are not a priority target for handling nutritional cases compared with other vulnerable groups. The aim of this study was to examine the factors associated with diet quality among adolescents in post-disaster areas in Indonesia. A cross-sectional study was performed with 375 adolescents aged 15–17 years, representing adolescents living close to the areas most affected by a significant disaster in 2018. The variables obtained include adolescent and household characteristics, nutritional literacy, healthy eating behavior constructs, food intake, nutritional status, physical activity, food security, and diet quality. The diet quality score was low, with only 23% of the total maximum score. Vegetables, fruits, and dairy scored the lowest, whereas animal protein sources scored the highest. Higher eating habits of animal protein sources; being healthy; normal nutritional status of adolescents; higher vegetable and sweet beverage norms of mothers; and lower eating habits of sweet snacks; animal protein sources; and carbohydrate norms of mothers are associated with higher diet quality scores in adolescents (*p* < 0.05). Improving the quality of adolescent diets in post-disaster areas needs to target adolescent eating behavior and changes in mothers’ eating behavior.

## 1. Introduction

Adolescents are a critical group in the manifestation of non-communicable diseases in adulthood; they provide an important contribution to nutritional improvement between generations [1]. Fulfillment of nutrition at this stage will have future impacts [2]. Appropriate diet quality is necessary for growth and the prevention of nutritional status-related macro- and micronutrient deficiencies or excess intakes [3].

Low diet quality is a major contributor to nutritional problems in low-middle-income countries (LMICs), where malnutrition remains a serious public health problem [1,4,5,6]. In Indonesia, the prevalence of underweight, stunting, and overweight adolescents aged 16–18 years reached 8.1%, 26.9%, and 13.5%, respectively, in 2018 [7]. The percentage of adolescent obesity increased by almost half from the previous year (7.3%), whereas the prevalence of underweight and stunting decreased to 19.4% and 31.2%, respectively, in 2013 [8].

Achieving good diet quality is difficult in LMICs, where starchy staple foods dominate diets, whereas the sources of animal foods, fruits, and vegetables are unavailable or difficult to obtain [9,10]. Other factors, including attitude, nutrition literacy, family support, friends or influential people for adolescents, and the ability to eat a balanced diet are also obstacles that hinder the achievement of good diet quality in adolescents [11,12,13].

In post-disaster areas, the diet quality of adolescents can be worse because this group is not a priority target for addressing nutritional cases, which typically focus on other vulnerable groups, such as toddlers and pregnant women [14]. Additionally, the food security of the family and the socioeconomic structure of the community have changed, thereby affecting the quality of the family’s diet, including that of adolescents [15].

In post-disaster areas, diet quality and its influencing factors have yet to be studied in detail. In contrast, interventions that focus on improving the quality of diets in the adolescent group in the post-disaster period need to be performed, particularly during rehabilitation and post-construction when individuals start living in normal conditions and determine the fulfillment of food in their respective households. This study aimed to determine the factors that influence diet quality in adolescents in post-disaster areas in Indonesia. The research results can be useful for designing nutrition and health programs for adolescents in post-disaster areas.

## 2. Materials and Methods

### 2.1. Study Population

From October 2021 to January 2022, a cross-sectional study was conducted on adolescents aged 15–17 years attending high school in the Indonesian city of Palu, which is located close to the area most affected by a major natural disaster in September 2018. The inclusion criteria were students in class X or XI, who lived with their mother, were willing to participate in the study, and signed an informed assent themselves and informed consent from their mother.

Sample determination was calculated on the basis of the formula [16], using 95% and 5% confidence and precision levels, respectively; the proportion used was 40.71%, which is the proportion of adolescents with vulnerable households. This proportion is used because the study sample is the subject of an initial De-Nulit study. The De-Nulit study is a study of nutritional literacy and diet quality in adolescents in food-insecure households in Indonesia. A total of 405 adolescents were randomly taken, and only 395 were successfully interviewed and had complete data.

### 2.2. Eating Habit and Construction of a Diet Quality Score

Adolescent food consumption includes eating habits of carbohydrates; vegetables; fruits; animal (including dairy) and plant protein sources; salty, sweet, and fatty foods; and sweet beverages, as assessed using a food frequency questionnaire. Answer scores were >1 time per day (score 5), 1 time per day (score 4), 3–6 times per week (score 3), 1–2 times per week (score 2), and <3 times per month (score 1) [7]. 

Diet quality in adolescents was assessed using the IGS3-60, which is the Healthy Eating Index developed for adolescents in Indonesia [17] and incorporates the iron component. The types of food consumed by the participants were grouped into carbohydrate foods, animal-based protein sources, plant-based protein sources, fruits, vegetables, dairy, and iron. All components in the diet quality assessment were food groups, except for iron. The inclusion of iron in the diet quality index is based on the fact that special attention needs to be addressed to the prevalence of anemia in adolescents in Indonesia, which is a moderate-level public health problem [7]. The average number of food portions was based on a 2-day non-consecutive 24-h food recall. Information on the type and amount of food intake was collected in household measurement and subsequently converted into grams using a food picture [18]. Modified IGS3-60 validation was performed by comparing the IGS value with the mean adequacy ratio. The correlation value was 0.82 (*p* < 0.01).

### 2.3. Other Covariates

The data collected included adolescent characteristics, such as age, and gender, nutritional knowledge, nutritional literacy, attitudes, subjective norms, behavioral control, intention to have a healthy diet, influence of friends, and parents, food consumption, diet quality, nutritional status, physical activity, and health conditions. The data obtained from mothers in the form of the socioeconomic conditions of adolescents and their families included household expenditures, mother’s educational level, household size, family type, knowledge of nutrition, maternal nutritional literacy, and maternal food norms, as well as food allocation in the household and food security.

Household expenditures were assessed as a proxy indicator of household income. Moreover, mother’s educational level, maternal nutritional literacy, household size, family type, food norms, and maternal food consumption habits were examined. Expenditures were categorized into quartiles. The mother’s educational level was divided into no school, basic education, secondary education, and higher education [19]. The maternal nutritional literacy was determined on the basis of the mean score of functional literacy, interactive literacy, and critical literacy components. The household size was divided into small, medium, and large [20]. The family type was divided into electron (the family consists of a father or a mother and unmarried children), nuclear (if a father, a mother, and unmarried children were in the family), atom (a father, a mother, unmarried children, and other unmarried family members), molecular (two married couples in different generations with or without family who are married or unmarried), and joint (two or more married couples in one generation or three or more couples in multi-generation) [21]. The mother’s eating norm was determined on the basis of the mean value of the Healthy Eating Norm [22]. Information on the Healthy Eating Norm was obtained from the question, “How often do you eat the following foods and drinks so that you can live a healthy life until you are old?” followed by a list of food groups classified on the basis of the balanced nutrition guidelines [22,23]. The Healthy Eating Norm response scale consisted of never, <3 times per month, 1–2 times per week, 3–6 times per week, 1 time per day, and >1 time per day [7]. Food allocation in households was assessed using a Likert scale question. Mothers were asked to rank each household member based on food allocation in the order from “more diverse” to “least diverse;” subsequently, it will be determined whether the adolescent is a priority or not a priority in family food allocation [24]. Food allocation consisted of carbohydrate and protein sources, vegetables, and fruits. Mothers’ eating habits were determined on the basis of the mean score for eating vegetables; fruits; animal (including diary) and plant protein sources; salty, sweet, and fatty foods; and sweet beverages measured using a food frequency questionnaire with a response scale of <3 times per month, 1–2 times per week, 3–6 times per week, 1 time per day, and >1 time per day [7]. Household food security was measured using the Household Food Insecurity Access Scale questionnaire consisting of nine questions [25] that were validated for adolescent households in Indonesia [26]. This variable was categorized into secure (0–1), slightly food insecure (2–7), moderate food insecure (8–14), and severe food insecure (15–27). 

The parents’ and peers’ influence was determined on the basis of the Social Support Scales scores [27]. The Social Support Scales consisted of 14 questions to assess the influence of parents and 11 questions to determine the influence of peers. The questionnaire was translated to Bahasa Indonesia and validated using Cronbach’s alpha >0.80. Nutrition literacy was assessed using a validated questionnaire (Cronbach’s alpha ≥ 0.70) that was modified from the Nutrition Literacy Inventory (NLI-28) [28]. The scoring was based on a Likert scale consisting of five choices, including “strongly agree,” “agree,” “undecided,” “disagree,” and “strongly disagree.” Each statement was scored from 1 point as the lowest to 5 points as the highest. The mean score was used in the statistical test.

The construction of eating behavior consisted of attitudes, subjective norms, behavioral control, and intentions to have a healthy diet. These Theory of Planned Behavior constructs on a healthy diet were assessed using a validated and reliability-tested questionnaire [29]. The scoring was based on five answer choices for each statement, such as “strongly agree,” “agree,” “undecided,” “disagree,” and “strongly disagree.” Responses to each positive statement were scored from 5 to 1 (strongly agree to disagree strongly), and negative statements were scored from 1 to 5 (strongly agree to disagree strongly). Attitudes, subjective norms, behavioral control, and intentions were determined on the basis of the mean score in the statistical analysis. 

Body image was determined using the Contour Drawing Rating Scale (CDRS) method [30]. The CDRS has been validated in Malaysian adolescents who are very close to Indonesian culture and body structure [30]. Participants were asked to choose one of the nine images that most closely resembled the current state of their body and their most desirable body image. Body image is a range of values for the desired and actual body shape. Values ranged from −8 (wants to be skinny) to 8 (wants to be fat).

To measure the body mass index (BMI) according to age, the nutritional status of adolescents assessed included weight and height. BMI is calculated by comparing weight (kilograms) with the square of height (meters). The BMI according to the age of adolescents was classified on the basis of the World Health Organization classification, which includes severe malnutrition (<−3 SD), thinness (−3 to <−2 SD), good nutrition (normal, −2 to +1 SD, over nutrition (overweight, +1 to +2 SD), and obesity (obese, >+2 SD) [31]. 

Physical activity was assessed using the adolescent’s physical activity level (PAL). Information on the participant’s physical activity was collected through a 24-h physical activity recall for two non-consecutive days. The average duration of the participant’s physical activity (hours) for 24 h multiplied by the physical activity ratio score for each activity refers to the FAO [32]. PALs of 1.40–1.69, 1.70–1.99, and 2.00–2.40 were categorized as light (light), moderate (moderate), and heavy (vigorous) activities, respectively. Health status was assessed by the number of days the participant was absent from school in a month. Participants were categorized as healthy if they had never been sick and had never been unable to attend school in the past month and were categorized as sick if they did not attend school at least one day because of illness.

### 2.4. Statistical Analysis

Data normality was identified using the Kolmogorov–Smirnov test and was found to be not normally distributed. However, each variable’s mean, standard deviation, and presentation are presented descriptively to provide comparable information with previous studies. The chi-square test and Kruskal–Wallis test were applied to assess the difference between gender, differences between adolescents eating habits, and mothers’ eating habits, and norms. The Spearman correlation test was used to inspect the correlation between the construction of eating behavior and diet quality and between adolescents’ eating habits, mothers’ eating habits, and mothers’ eating norms.

To examine factors related to adolescent diet quality, a logistic regression analysis was performed. The diet quality score as the dependent variable was divided into two categories based on the mean score. To examine the diet quality score based on gender and nutritional status after removing participants with a ratio of energy intake and basal metabolic rate below 0.9, a sensitivity analysis was performed [33]. In the process of performing logistic regression analysis, re-coding was performed on several variables because it has a high error standard after analysis with initial coding. The variables included adolescents’ eating habits, mothers’ eating habits, and mothers’ eating norms. The frequencies of eating <3 times a month, 1–2 times a week, and 3–6 times a week were combined into one category, whereas the other frequencies remained. Analysis was performed using SAS, and the *p*-value of statistical significance was <0.05.

## 3. Results

A total of 395 adolescents were included in this study, with 66.3% and 33.77% female and male participants, respectively. Most adolescents were living in small households (80.3%), with nuclear families (51.4%) being the major family type. The average expenditure in an adolescent family was 2.4 million rupiahs, with the educational levels of mothers dominated by elementary education graduates (43.8%). Thirty-nine percent of adolescents were living in food-secure households. The rest were adolescents who were living in households with mild-to-severe food insecurity. Adolescents were a family priority in the allocation of food (>78%). Furthermore, most adolescents had a normal nutritional status (77.5%), with a mild activity level (52.7%) and a body image of feeling fat or wanting to be skinny (57.5%). No difference was observed between gender characteristics except the physical activity level. Female participants were more sedentary (95.5%) than male participants (78.2%) (Table 1).

**Figure 1:**
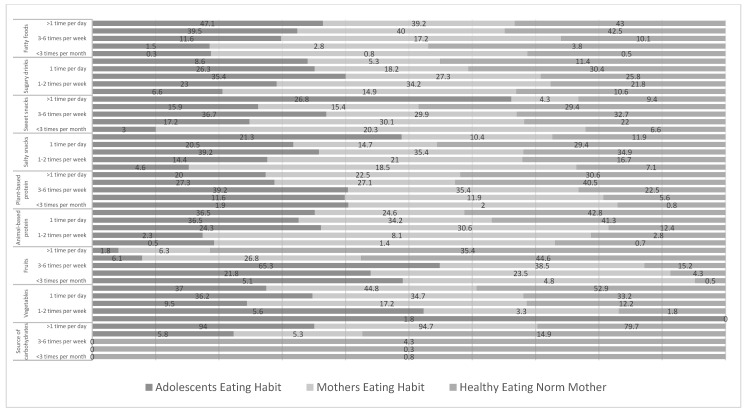
Adolescents’ eating habits, mothers’ eating habits, and mothers’ healthy eating norms.

Adolescents’ eating habits differ from their mothers’, except for carbohydrate and plant-based protein sources. More than 90% of mothers and children consumed carbohydrate sources more than once a day, whereas plant-based protein sources were most frequently consumed only 3–6 times a week (>35%). Adolescents more frequently consumed animal protein sources as well as sweet snacks, sweet beverages, salty snacks, and fatty foods than their mothers (*p* < 0.05). In contrast, mothers more frequently consumed vegetables and fruits than adolescents (*p* < 0.05) (Figure 1, Table 2). A significant positive correlation between adolescents’ and mothers’ eating habits was noted (*p* < 0.05), except for the habit of eating sweet snacks, which was observed to have no correlation between adolescents’ and mothers’ eating habits (Table 2).

Compared with eating norms, a significant difference between the mother’s eating habits and her eating norms, as well as the mother’s eating norms and the adolescent’s eating habits was noted (Table 2). Maternal norms were higher, particularly in the eating habits of vegetables, fruits, and animal, and vegetable protein sources, than adolescent eating habits. No difference was observed between the norms of drinking sweets, salty snacks, and fatty foods between the adolescents’ eating habits and the mothers’ eating norms. However, a positive correlation was noted between mothers’ eating norms and mothers’ and adolescents’ eating habits for all food components (*p* < 0.05). Only the eating habits of carbohydrate sources showed no correlation between mothers’ eating norms and mothers’ and adolescents’ eating habits (*p* > 0.05).

From the behavior-forming constructs, healthy eating behavior was positively correlated with attitudes and subjective eating norms. However, unhealthy eating behavior had a negative correlation with intention. Healthy and unhealthy eating behaviors were correlated (r = 0.46) and positively related to the dietary quality (Table 3).

The adolescents’ food intakes were less than the recommended daily portions. Only protein-based animal dishes had an intermediate portion close to the recommended daily portion (Table 4). Moreover, the mean total score of the diet quality was low, with only 16 of the maximum score of 70. Vegetables, fruits, and dairy scored lower, with average scores of 0.0, 0.5, and 0.7, respectively. The highest score was on a protein-based animal dish, with a score of 5.8 of the maximum score of 10. 

After removing more than 50% of adolescents with underreporting energy, the diet quality score was higher by five points. Males had significantly higher scores than females (*p* < 0.05) (Table 4). The change was mainly seen in the iron score, which was much higher for males than females. Iron intake in males meets the Estimated Average Requirements (EAR) but not in females.

Additionally, the diet quality score was significantly higher in the obese group than that in the normal group when presenting on the basis of nutritional status (*p* < 0.05) (Table 5). However, the difference between the obese and normal groups was only observed in female participants. Considering the underreporting group, it was observed that the diet quality score was not different between the nutritional status group in female and male participants. 

Binary logistic regression analysis included variable participant characteristics and behavior components, revealing that diet quality was associated with adolescent functional nutrition literacy, health status, nutritional status, and eating habits of animal-based protein sources (*p* < 0.05). Mothers’ eating habits and norms, including sweet beverages, sweet snacks, and animal-based protein sources, as well as mothers’ eating norms of carbohydrates and vegetables were related to the adolescents’ diet quality (*p* < 0.05). Adolescents with higher functional nutrition literacy, healthy, and eating animal-based protein sources more frequently—with mothers consuming sweet beverages and high norms of vegetables—were associated with higher diet quality (*p* < 0.05). Conversely, obese adolescents with mothers who preferred to eat animal protein and sweet snacks less frequently and had a low norm of eating carbohydrates were associated with lower diet quality (*p* < 0.05) (Table 6).

## 4. Discussion

The aim of this study was to identify factors related to the quality of adolescent diets in post-disaster areas. The quality score of adolescents in this study was low, with only 23% of the total maximum score. Some food group scores have scores below one, including vegetables, fruits, and dairy. Furthermore, certain conditions that were more vulnerable to food shortages, including conflict areas, show similar results [34,35]. However, in this study, we observed that the scores of animal-based protein sources were higher than those of vegetable, fruit, or carbohydrate sources. The results of our study are in contrast with those of other studies that reported that fruit and vegetable intake was higher than that of animal protein sources in developing countries; however, their vegetable and fruit intake also did not fulfill the recommended value [36,37]. Low animal food intake in vulnerable conditions is associated with low availability of animal food sources [38]. However, in this study, the adolescents live close to the sea; therefore, the geography of the place makes animal-based protein sources derived from the sea, including fish, easy to obtain and favored by the adolescents [39,40]. 

In this study, the diet quality score was lower than that of most studies, except for the study in Brazil [41]. Compared with our study, the mean adolescent diet quality score in the urban areas of the Indonesian capital was 33% or above 10 points [42]. In contrast, in urban Malaysia, the mean diet quality score was much higher, at 56% [43], which is similar to the quality of diets in some developed countries [44,45]. Analysis involving adolescents without underreporting also showed that the diet quality score in this post-disaster area was low (31%), close to the diet quality score of adolescents in urban Indonesia [42]. Females had lower scores than males, which also agrees with the results of other studies [41,42].

Adolescent food habits also have a significant role in the quantity of adolescent food intake. However, we observed that the high consumption of adolescents does not necessarily indicate a high score on the dietary quality score of carbohydrate-source foods consumed more often than animal-source foods. The high frequency of food consumption is only occasionally positively correlated with dietary quality [46]. Adolescents can often consume certain food groups. However, portions cannot meet the recommended values; therefore, quantitatively, the amount of food intake needs to be adequate [47]. 

In this study, a positive correlation was noted between adolescents’ and mothers’ eating habits, as well as adolescents’ eating habits and mothers’ eating norms. Adolescents’ eating habits are related to the mothers’ eating habits and inherent eating norms [48,49]. The largest correlation was observed in fatty eating habits and salty snacks, with adolescents eating more frequently than their mothers. Moreover, several previous studies have stated a correlation between adolescent eating habits and maternal eating norms, particularly in the low eating habits of vegetables and fruits and the high consumption of sweet, salty, and fatty foods [50,51,52]. The trend of fatty and salty foods is rapidly increasing in developing countries [53]. With the development of food technology that produces packaged foods, the variety of processed snack foods has mushroomed to remote areas, causing individuals on the edge of the city to acquire high access to snack foods [53]. Since rehabilitation, the community’s condition has gradually improved in the post-disaster area; therefore, economic growth has returned to normal. Trade, including ultra-processing food and street food, is expanding again.

Furthermore, adolescents’ eating habits are influenced by factors that shape eating behavior, including attitudes, subjective norms, and behavioral control. We observed that subjective norms were positively correlated with positive and negative eating habits. The influence of other individuals is related to positive and negative eating habits in adolescents [54]. The support of others is indispensable to increasing self-confidence and self-efficacy [55]. In this study, the intention was negatively correlated with negative eating habits. In contrast, a positive although insignificant correlation was noted between adolescents’ positive intentions and eating habits. Something similar was noted in studies of food-insecure adolescents [56]. Adolescents’ intentions predict behavior in performing something, particularly if it is followed by adolescent environmental support, such as good food availability and access [57]. 

In this study, eating habits, and behavior constructs, such as attitudes, and subjective norms, were positively correlated with adolescents’ dietary quality. However, the association between the construction of behavior changes and diet quality diminished after being controlled for other variables in the regression test. Simultaneously, the eating habits of animal-based protein sources became significantly positively correlated. Protein is a significant component of the daily diet and is necessary for normal growth and development in adolescents [58]. Compared with other food sources, animal-based food sources have the highest total dietary quality scores. This relationship suggests that animal-based food sources contribute to the high-quality value of the diet. Similar to previous studies, animal protein sources’ contribution to dietary quality is 60% [59]. However, the value of the animal protein source score still needs to reach the maximum recommended score. Additionally, the intake of other food groups remains less than that of animal protein sources; therefore, it only slightly contributes to the quality score of the adolescent diet. 

Other factors that were observed to have an association with adolescent diet quality after adjusting for other variables include eating sweet snacks and mothers’ norms of eating carbohydrates and vegetables. Eating sweet snacks and mothers’ norms of eating carbohydrate sources were observed to be negatively related to adolescents’ diet quality. In contrast, mothers’ norms of eating vegetables were positively associated with adolescents’ diet quality. A mother’s eating habits can arise from her norms and subsequently be followed by the adolescent; therefore, it becomes their habit [60]. Mothers’ eating habits set an example for adolescents to emulate [49]. Conversely, mothers may not be used to eating certain foods, such as sweet snacks, or vegetables. However, high food norms influence mothers to provide greater access for adolescents to obtain these foods for consumption [61].

Furthermore, the regression test revealed that mothers’ sweet drinking habits were positively related to adolescent diet quality scores, and mothers’ animal-based protein source food eating norms were significantly negatively related. We observed an interaction effect between the food norms of mothers in animal protein sources and household food security status. Likewise, mothers’ sweet drinking habits were observed to interact with the habit of eating sweet snacks. Advanced analysis by performing a separate analysis based on the effect of the interactions noted could not be performed because of the small sample size. 

Other diet quality-associated variables were functional nutritional literacy, adolescent health status, and nutritional status. Functional nutrition literacy is basic literacy that is the foundation for higher-level literacy, such as interactive nutrition literacy and critical nutrition literacy [62]. A person’s ability to understand nutritional messages and information and an understanding of balanced nutrition helps adolescents choose the foods that must be ingested to improve the quality of their diet [63,64]. 

In adolescents who are not sick, the diet quality is known to be better than that of sick adolescents. In sick conditions, there is a tendency to choose bland foods owing to changes in appetite due to physiological influences [65,66]. The nutritional status being negatively related to dietary quality is also because of adolescents’ tendency for monotonous food selection [67]. In obese adolescents, eating is dominated by high-energy foods, including fatty foods [68]. Our study shows that obese adolescents have higher but insignificant diet quality scores in fruits and lower diet quality scores in all other food components. However, this result should be cautiously interpreted since we also noted that obese adolescents underreport their intake more than other nutritional status intakes in this study. We performed a sensitivity analysis. However, the number of obese adolescents decreased by more than half; therefore, we could not determine the total mean habitual intake of obese adolescents.

This study provides an overview of the diet quality of adolescents in vulnerable post-disaster areas who need more attention to efforts to improve their nutrition and health. We have included various factors that could affect diet quality in this study. However, variables still need to be fully covered, including the availability of food in the household and the preferences of the mother in food preparation. This study has yet to reach out to adolescents who are not in school and may have different eating habits and other factors related to the diet quality of adolescents who are in school.

## 5. Conclusions

Eating habits, health status, and nutritional status are factors that are related to the diet quality of adolescents. Moreover, mothers’ eating habits and norms are related to the diet quality of adolescents in post-disaster areas. In addition to adolescents, improving the diet quality of adolescents in post-disaster areas needs to target changes in mothers’ eating behavior.

## Figures and Tables

**Table 1:** Sociodemographic characteristics of adolescents.

**Characteristics**	**Overall** **(*n* = 395)**		**Males** **(*n* = 133)**		**Females** **(*n* = 262)**		***p*-Value ^†^**
	***n***	**%**	***n***	**%**	***n***	**%**	
**Age of adolescents (years)**							
15	160	40.5	48	36.1	112	42.7	0.39
16	173	43.8	61	45.9	112	42.7	
17	62	15.7	24	18.0	38	14.5	
**Household expenses**							
Quartile 1	99	25.1	39	29.3	60	22.9	0.19
Quartile 2	99	25.1	25	18.8	74	28.2	
Quartile 3	99	25.1	35	26.3	64	24.4	
Quartile 4	98	24.8	34	25.6	64	24.4	
**Household size**							
Small household	317	80.3	112	84.2	205	51.9	0.23
Medium household	65	16.5	16	12.0	49	18.7	
Large household	13	3.3	5	3.8	8	3.1	
**Family type**							
Electron family	38	9.6	12	9.0	26	9.9	0.99
Nuclear family	204	51.6	70	52.6	134	51.1	
Atom family	78	19.7	27	20.3	51	19.5	
Molecular family	52	13.2	16	12.0	36	13.7	
Joint family	23	5.8	8	6.0	15	5.7	
**Mother’s educational level**							
No school	10	2.5	6	4.5	4	1.5	0.05
Basic education	173	43.8	56	42.1	117	44.7	
Secondary education	167	42.3	50	37.6	117	44.7	
Higher education	45	11.4	21	15.8	24	9.2	
**Food allocation in the household**							
**Carbohydrate sources**							
Adolescent is a priority	311	78.7	106	79.7	205	78.2	0.74
Adolescent is not a priority	84	21.3	27	20.3	57	21.8	
**Animal-based protein sources**							
Adolescent is a priority	309	78.2	108	81.2	201	76.7	0.31
Adolescent is not a priority	86	21.8	25	18.8	61	23.3	
**Plant-based protein sources**							
Adolescent is a priority	315	79.7	108	81.2	207	79.0	0.70
Adolescent is not a priority	80	20.3	25	18.8	55	21.0	
**Vegetables**							
Adolescent is a priority	313	79.2	112	84.2	201	76.7	0.08
Adolescent is not a priority	82	20.8	21	15.8	61	23.3	
**Fruits**							
Adolescent is a priority	313	79.2%	110	82.7	203	77.5	0.23
Adolescent is not a priority	82	20.8	23	17.3	59	22.5	
**Household food security**							
Secure	154	39%	56	42.1	98	37.4	0.09
Slightly food insecure	64	16.2%	14	10.5	50	19.1	
Moderate food insecure	104	27,1%	34	25.6	73	27.9	
Severe food insecure	70	17.7%	29	21.8	41	15.6	
**Physical activity**							
Very light	208	52.7	48	36.1	160	61.1	0.00 *
Light	146	37	56	42.1	90	34.4	
Moderate	28	7.1	20	15.0	8	3.1	
Vigorous	13	3.3	9	6.8	4	1.5	
**Nutritional status**							
Severe thinness	15	3.8	7	5.3	8	3.1	0.14
Thinness	31	7.8	15	11.3	16	6.1	
Normal	306	77.5	93	69.9	213	81.3	
Overweight	26	6.6	11	8.3	15	5.7	
Obese	17	4.3	7	5.3	10	3.8	
**Body image**							
Feeling fat	227	57.5	81	60.9	146	55.7	0.61
Normal	56	14.2	17	12.8	39	14.9	
Feeling thin	112	28.4	35	26.3	77	29.4	

† *p*-value from the Chi-square test to see significant differences between male and female; * Significant difference between male and female participants using the Chi-square test (*p* < 0.05).

**Table 2:** Correlation between adolescents’ eating habits, mothers’ eating habits, and mothers’ healthy eating norms.

Variable	Mean (SD)		
	Adolescents’ Eating Habits Mean	Mothers’ Eating Habits	Mothers’ Healthy Eating Norms
Carbohydrate sources	4.9 (0.2)	4.9 (0.2)	4.7 (0.7) *^#^
Vegetables	4.0 (1.0)	4.2 (0.8) *^r^	4.4 (0.8) *^#rrR^
Fruits	2.8 (0.7)	3.1 (1.0) *^r^	4.1 (0.9) *^#rR^
Animal-based protein sources	4.1 (0.9)	3.7 (1.0) *^r^	4.2 (0.8) *^#rR^
Plant-based protein sources	3.5 (1.0)	3.5 (1.1) ^r^	3.9 (0.9) *^#rrR^
Salty snacks, mean (SD)	3.4 (1.1)	2.8 (1.2) *^r^	3.2 (1.1) ^rrR^
Sweet snacks, mean (SD)	3.5 (1.2)	2.5 (1.1) *	3.1 (1.1) *^#rrR^
Sugary drinks, mean (SD)	3.1 (1.1)	2.6 (1.4) *^r^	3.1 (1.2) ^rrR^
Fatty foods, mean (SD)	4.3 (0.8)	4.1 (1.8) *^R^	4.2 (0.8) ^rR^

* Significant difference with adolescents’ eating habits (*p* < 0.05), ^#^ Significant difference with mothers’ eating habits (*p* < 0.05), ^r^ Significant correlation compared with adolescents’ eating habits r ≤ 0.50 (*p* < 0.05), ^R^ Significant correlation compared with adolescents’ eating habits r > 0.50 (*p* < 0.05), ^r r^ Significant correlation compared with mothers’ eating habits r ≤ 0.50 (*p* < 0.05).

**Table 3:** Correlation between eating behavior constructs based on the Theory of Planned Behavior and diet quality among adolescents.

Variable	Diet Quality Score	Healthy Eating Behavior	Unhealthy Eating Behavior	Intention	Attitude	Subjective Norm	Control Behavior
Diet quality score	1	0.09	0.15 *	0.07	0.14 *	0.12 *	0.08
Healthy eating behavior	0.09	1	0.46 *	0.04	0.14 *	0.14 *	0.14
Unhealthy eating behavior	0.15 *	0.46 *	1	−0.13 *	0.18 *	0.19 *	0.05
Intention	0.07	0.04	−0.13 *	1	0.03	0.08	0.03
Attitude	0.14 *	0.14 *	0.18 *	0.03	1	0.53 *	0.50 *
Subjective norm	0.12 *	0.14 *	0.19 *	0.08	0.53 *	1	0.47 *
Control behavior	0.08	0.14	0.05	0.03	0.50 *	0.47 *	1
Mean (SD)	16 (9.49)	3.9 (0.5)	3.6 (0.5)	3.6 (0.5)	3.7 (0.4)	3.6 (0.4)	3.4 (0.4)

* *p* < 0.05 based on Spearman’s correlation test.

**Table 4:** Food intake, portions, and diet quality score in the adolescents.

No	Component	Food Intake (Gram/Day)			Portion (Portion/Day)				Diet Quality Score			
		Males	Females	Overall	Males	Females	Overall	Recommendation	Males	Females	Overall	Maximum Recommended Score
*n* = 395 (males, *n* = 133; females, *n* = 262)												
1	Carbohydrate group	402 (156)	324 (125) *	350 (141)	4.0 (1.6)	3.2 (1.2) *	3.5 (1.4)	5.0 (females), 8.0 (males)	2.8 (2.8)	4.2 (2.9) *	3.8 (2.9)	10.0
2	Vegetables	43 (37)	34 (36) *	37 (36)	0.4 (0.4)	0.3 (0.4) *	0.4 (0.4)	4.0	0.1 (0.6)	0.0 (0.3)	0.0 (0.4)	10.0
3	Fruits	18 (46)	28 (75)	24 (67)	0.4 (0.9)	0.6 (1.5)	0.5 (1.3)	3.0	0.4 (1.5)	0.6 (1.9)	0.5 (1.8)	10.0
4	Animal-based protein sources	115 (70)	111 (81)	112 (78)	2.9 (2.2)	2.7 (1.9)	2.8 (2.0)	3.0	6.1 (3.8)	5.6 (4.0)	5.8 (3.9)	10.0
5	Plant-based protein sources	78 (116)	70 (106)	73 (109)	1.6 (2.3)	1.4 (2.1)	1.5 (2.2)	3.0	2.5 (3.8)	2.3 (3.9)	2.4 (3.8)	10.0
6	Dairy	14 (57) ^#^	24 (69) ^#^	21 (66) ^#^	0.1 (0.3) ^#^	0.1 (0.3) ^#^	0.1 (0.3) ^#^	1.0^#^	0.5 (1.8)	0.8 (2.3)	0.7 (2.1)	10.0
7	Iron	8.7 (5.0) ^##^	8.4 (5.8) ^##^	8.5 (5.4) ^##^	8.7 (5.0) ^##^	8.5 (5.8) ^##^	8.5 (5.5) ^##^	15.0 (females), 11.0 (males) ^##^	3.4 (4.1)	2.6 (3.4)	2.9 (3.7)	10.0
Total									15.6 (9.5)	16.2 (9.5)	16.0 (9.5)	70.0
*n* = 172 (males, *n* = 37; females, *n* = 135)												
1	Carbohydrate group	511 (170)	377 (126) *	406 (147)	5.1 (1.7)	3.7 (1.3) *	4.1 (1.5)	5.0 (females), 8.0 (males)	4.6 (2.2)	5.1 (2.8)	5.0 (2.6)	10.0
2	Vegetables	54 (45)	39 (36)	42 (38)	0.5 (0.4)	0.4 (0.4)	0.4 (0.4)	4.0	0.3 (1.1)	0.0 (0.4)	0.1 (0.7)	10.0
3	Fruits	17 (39)	36 (94)	32 (86)	0.4 (0.8)	0.7 (1.9)	0.6 (1.7)	3.0	0.4 (1.4)	0.8 (2.2)	0.7 (2.1)	10.0
4	Animal-based protein sources	145 (91)	131 (96)	134 (95)	3.5 (2.2)	3.3 (2.4)	3.3 (2.3)	3.0	6.8 (3.4)	6.4 (4.0)	6.5 (3.8)	10.0
5	Plant-based protein sources	153 (152)	100 (128) *	111 (134)	3.1 (3.0)	2.0 (2.6) *	2.2 (2.7)	3.0	5.0 (4.6)	3.5 (4.40)	3.8 (4.4)	10.0
6	Dairy	32 (96)	32 (82)	32 (85.4)	0.2 (0.5)	0.2 (0.4)	0.2 (0.4)	1.0^#^	1.0 (2.8)	1.1 (2.6)	1.1 (2.7)	10.0
7	Iron	13.2 (5.1)	11.2 (6.5) *	11.6 (6.3)	13.2 (5.1)	11.2 (6.5) *	11.6 (6.3)	15.0 (females), 11.0 (males) ^##^	7.0 (4.0)	4.1 (3.6)	4.7 (3.9)	10.0
Total									24.7 (8.1)	21.1 (9.0) *	21.9 (8.9)	70.0

^#^ millilitter, ^##^ milligram, * Significant difference between male and female participants (*p* < 0.05) based on the Kruskal–Wallis test.

**Table 5:** Diet quality scores based on the nutritional status in the adolescents.

Nutritional Status	Component Diet Quality							
	Carbohydrate Group	Vegetables	Fruits	Animal-Based Protein Sources	Plant-Based Protein Sources	Dairy	Iron Nutrient (mg)	Total
*n* = 395								
Males (*n* = 133)								
Severe thinness (*n* = 7)	2.9 (3.9)	0.00 (0.00)	0.00 (0.00)	4.3 (4.5)	1.4 (2.4)	1.4 (3.8)	2.1 (3.9)	12.1 (10.4)
Thinness (*n* = 15)	3.0 (2.5)	0.00 (0.00)	1.00 (2.07)	5.7 (4.2)	1.0 (2.1)	0.0 (0.0)	4.0 (4.3)	14.7 (7.7)
Normal (*n* = 93)	2.8 (2.8)	0.11 (0.73)	0.38 (1.52)	6.3 (3.8)	2.7 (4.0)	0.5 (1.8)	3.6 (4.1)	16.2 (9.9)
Overweight (*n* = 11)	3.2 (2.5)	0.00 (0.00)	0.00 (0.00)	6.4 (2.3)	3.6 (4.5)	0.5 (1.5)	3.1 (4.6)	16.8 (11.0)
Obese (*n* = 7)	1.4 (2.4)	0.00 (0.00)	0.00 (0.00)	5.7 (4.5)	2.1 (2.7)	0.0 (0.0)	2.1 (2.7)	11.3 (3.8)
Females (*n* = 262)								
Severe thinness (*n* = 8)	5.0 (2.7)	0.00 (0.00)	2.50 (3.78)	6.9 (3.7)	0.0 (0.0)	0.0 (0.0)	0.6 (1.8)	15.0 (4.6)
Thinness (*n* = 16)	3.1 (3.1)	0.00 (0.00)	0.31 (1.23)	4,7 (4.3)	1.6 (3.0)	0.9 (2.7)	1.3 (2.9)	11.9 (8.1)*
Normal (*n* = 213)	4.4 (2.9)	0.02 (0.34)	0.54 (1.83)	5.6 (4.0)	2.6 (4.0)	0.8 (2.3)	2.8 (3.4)	16.8 (9.8)
Overweight (*n* = 15)	3.3 (3.1)	0.00 (0.00)	0.67 (1.76)	6.7 (4.1)	2.3 (4.2)	1.3 (3.0)	2.0 (3.2)	16.3 (7.9)
Obese (*n* = 10)	3.0 (2.6)	0.00 (0.00)	1.00 (3.16)	3.5 (3.4)	0.0 (0.0)	1.0 (2.1)	1.5 (3.4)	10.0 (6.2)*
Overall								
Severe thinness (*n* = 15)	4.0 (3.4)	0.00 (0.00)	1.33 (3.0)	5.7 (4.2)	0.7 (1.8)	0.7 (2.6)	1.3 (3.0)	13.7 (7.7)
Thinness (*n* = 31)	3.1 (2.8)	0.00 (0.00)	0.65 (1.7)	5.2 (4.2)	1.3 (2.6)	0.5 (2.0)	2.6 (3.8)	13.4 (7.9)
Normal (*n* = 306)	3.9 (2.9)	0.05 (0.49)	0.49 (1.7)	5.9 (3.9)	2.6 (4.0)	0.7 (2.1)	3.0 (3.7)	16.7 (9.8)
Overweight (*n* = 26)	3.3 (2.8)	0.00 (0.00)	0.38 (1.4)	6.5 (3.4)	2.9 (4.3)	1.0 (2.5)	2.5 (3.8)	16.5 (9.1)
Obese (*n* = 17)	2.4 (2.6)	0.00 (0.00)	0.59 (2.4)	4.4 (3.9)	0.9 (2.0)	0.6 (1.7)	1.8 (3.0)	10.6 (5.3)*^#^
*n* = 172								
Males (*n* = 37)								
Severe thinness (*n* = 2)	7.5 (3.5)	0.00 (0.00)	0.00 (0.00)	2.5 (3.5)	0.0 (0.0)^#^	5.0 (7.1)	5.0 (7.1)	20.0 (14.1)
Thinness (*n* = 5)	5.0 (0.0)	0.00 (0.00)	1.00 (2.23)	6.0 (4.2)	2.0 (2.7)^#^	0.0 (0.0)	7.0 (4.5)	21.0 (6.5)
Normal (*n* = 27)	4.3 (2.3)	0.37 (1.33)	0.37 (1.33)	7.4 (3.2)	5.4 (4.6)	0.7 (2.7)	6.9 (4.0)	25.0 (8.1)
Overweight (*n* = 3)	5.0 (0.0)	0.00 (0.00)	0.00 (0.00)	5.0 (0.0)	10 (0.0)	1.7 (2.9)	10.0 (0.0)	31.7 (2.9)
Obese (*n* = 0)	-	-	-	-	-	-	-	-
Females (*n* = 135)								
Severe thinness (*n* = 5)	6.0 (2.2)	0.00 (0.00)	3.00 (4.47)	6.0 (4.2)	0.0 (0.0)	0.0 (0.0)	1.0 (2.2)	16.0 (5.5)
Thinness (*n* = 7)	5.0 (2.9)	0.00 (0.00)	0.71 (1.89)	4.3 (4.5)	2.9 (3.9)	1.4 (3.8)	2.1 (3.9)	16.4 (8.5)
Normal (*n* = 111)	5.2 (2.8)	0.05 (0.48)	0.72 (2.12)	6.7 (3.9)	3.8 (4.4)	1.0 (2.5)	4.5 (3.5)	21.9 (9.2)
Overweight (*n* = 9)	3.9 (3.3)	0.00 (0.00)	1.11 (2.21)	6.1 (4.2)	3.9 (4.9)	2.2 (3.6)	3.3 (3.5)	20.6 (6.8)
Obese (*n* = 3)	5.0 (0.0)	0.00 (0.00)	0.00 (0.00)	5.0 (5.0)	0.0 (0.0)	0.0 (0.0)	3.3 (5.8)	13.3 (10.4)
Overall (*n* = 172)								
Severe thinness (*n* = 7)	6.4 (2.4)	0.00 (0.00)	2.14 (3.93)	5.0 (4.1)	0.0 (0.0) *^#^	1.4 (3.8)	2.1 (3.9)	17.1 (7.6)
Thinness (*n* = 12)	5.0 (2.1)	0.00 (0.00)	0.83 (1.95)	5.0 (4.3)	2.5 (3.4)	0.8 (2.9)	4.2 (4.7)	18.3 (7.8)
Normal (*n* = 138)	5.0 (2.7)	0.11 (0.73)	0.65 (1.99)	6.8 (3.8)	4.1 (4.5)	1.0 (2.6)	4.9 (3.7)	22.5 (9.1)
Overweight (*n* = 12)	4.2 (2.9)	0.00 (0.00)	0.83 (1.95)	5.8 (3.6)	5.4 (5.0)	2.1 (3.3)	5.0 (4.3)	23.3 (7.8)
Obese (*n* = 3)	5.0 (0.0)	0.00 (0.00)	0.00 (0.00)	5.0 (5.0)	0.0 (0.0)	0.0 (0.0)	3.3 (5.8)	13.3 (10.4)

* Significant difference compared with normal nutritional status (*p* < 0.05), # Significant difference compared with overweight nutritional status (*p* < 0.05) based on the Kruskal–Wallis test.

**Table 6:** Logistic regression model of the relationship between diet-related behaviors and other characteristics with the diet quality score.

Variables	OR	95% CI		*p*-Value
**Age of adolescents**	0.63	0.39	1.01	0.06
**Gender of adolescents**				
Females	Ref			
Males	0.88	0.37	2.07	0.77
**Household expenses**				0.47
Quartile 1	Ref			
Quartile 2	1.31	0.50	3.47	0.59
Quartile 3	1.45	0.52	4.03	0.48
Quartile 4	0.67	0.21	2.16	0.51
**Household size**				0.74
Small household	Ref			
Medium household	0.70	0.21	2.35	0.57
Large household	1.41	0.13	15.49	078
**Family type**				0.63
Joint family	Ref			
Molecular family	0.70	0.11	4.59	0.71
Atom family	1.87	0.24	14.40	0.55
Nuclear family	1.02	0.13	8.10	0.99
Electron family	1.03	0.09	11.42	0.98
**Mother’s educational level**				0.96
No school	Ref			
Basic education	1.81	0.20	16.35	0.60
Secondary education	1.74	0.18	17.05	0.64
Higher education	1.90	0.16	22.32	0.61
**Maternal nutrition literacy**				
Functional nutrition literacy	0.73	0.32	1.64	0.45
Interactive nutrition literacy	1.47	0.61	3.54	0.40
Critical nutrition literacy	0.76	0.22	2.60	0.66
**Adolescents’ nutrition literacy**				
Functional nutrition literacy	2.89	1.29	6.45	0.01*
Interactive nutrition literacy	0.94	0.48	1.84	0.85
Critical nutrition literacy	0.85	0.32	2.21	0.74
**Food allocation in the household**				
**Carbohydrates**				
Adolescent is not a priority	Ref			
Adolescent is a priority	0.84	0.23	3.11	0.80
**Animal-based protein sources**				
Adolescent is not a priority	Ref			
Adolescent is a priority	2.14	0.20	22.89	0.53
**Plant-based protein sources**				
Adolescent is not a priority	Ref			
Adolescent is a priority	1.72	0.10	30.67	0.71
**Vegetables**				
Adolescent is not a priority	Ref			
Adolescent is a priority	1.24	0.09	17.02	0.87
**Fruits**				
Adolescent is not a priority	Ref			
Adolescent is a priority	1.08	0.09	13.44	0.95
**Household food security**				0.62
Secure	Ref			
Slightly food insecure	0.59	0.21	1.71	0.59
Moderate food insecure	1.05	0.40	2.76	0.92
Severe food insecure	1.39	0.43	4.55	0.59
**Mother’s eating habit**				
**Carbohydrates**				
Once a day	Ref			
>1 time a day	2.95	0.58	14.90	0.19
**Vegetables**				0.78
<3 times a week	Ref			
3–6 times a week	0.54	0.06	4.50	0.57
Once a day	0.43	0.04	4.15	0.46
>1 time a day	0.33	0.03	3.35	0.35
**Fruits**				0.14
<3 times a month	Ref			
1–2 times a week	1.70	0.26	11.21	0.58
3–6 times a week	1.22	0.17	8.95	0.85
Once a day	3.33	0.37	29.62	0.28
>1 time a day	6.74	0.64	70.46	0.11
**Animal-based protein sources**				0.02*
<1 time a day	Ref			
Once a day	1.38	0.55	3.44	0.49
>1 time a day	0.18	0.04	0.71	0.01
**Plant-based protein sources**				0.11
<1 time a day	Ref			
Once a day	0.44	0.16	1.19	0.11
>1 time a day	0.34	0.10	1.12	0.08
**Sweet snacks**				0.01*
<3 times a month	Ref			
1–2 times a week	1.92	0.61	6.05	0.27
3–6 times a week	0.30	0.08	1.08	0.06
Once a day or more	0.67	0.73	3.02	0.67
**Sweet beverages**				0.02*
<3 times a month	Ref			
1–2 times a week	6.74	1.77	25.7	0.06
3–6 times a week	9.17	2.23	37.8	0.00
Once a day or more	5.45	1.22	24.3	0.03
**Salty foods**				0.14
<3 times a month	Ref			
1–2 times a week	1.51	0.43	5.30	0.52
3–6 times a week	1.31	0.37	4.66	0.68
Once a day	0.67	0.13	3.36	0.63
>1 time a day	8.91	1.11	71.43	0.04
**Fatty foods**				0.17
<1 time a day	Ref			
Once a day	0.89	0.29	2.74	0.84
>1 time a day	0.29	0.07	1.16	0.08
**Mother’s eating norm**				
**Carbohydrates**				0.03*
<1 time a day	Ref			
Once a day	0.09	0.01	0.61	0.01
>1 time a day	0.09	0.01	0.54	0.01
**Vegetables**				0.01*
<1 time a day	Ref			
Once a day	3.20	0.77	13.37	0.11
>1 time a day	13.39	2.43	73.68	0.00
**Fruits**				0.13
<1 time a day	Ref			
Once a day	0.48	0.15	1.55	0.22
>1 time a day	0.25	0.07	0.97	0.05
**Animal-based protein sources**				0.55
<1 time a day	Ref			
Once a day	0.53	0.15	1.86	0.32
>1 time a day	0.82	0.17	3.88	0.80
**Plant-based protein sources**				0.82
<1 time a day	Ref			
Once a day	0.84	0.32	2.21	0.73
>1 time a day	1.27	0.30	5.28	0.74
Sweet foods				0.48
<3 times a month	Ref			
1–2 times a week	1.88	0.29	12.04	0.51
3–6 times a week	0.89	0.13	6.21	0.90
Once a day	1.15	0.15	8.70	0.89
>1 time a day	3.49	0.31	38.90	0.31
**Sweet beverages**				0.06
<3 times a month	Ref			
1–2 times a week	0.28	0.05	1.46	0.13
3–6 times a week	0.69	0.13	3.57	0.66
Once a day	0.19	0.03	1.12	0.07
>1 time a day	0.14	0.02	0.92	0.04
**Salty foods**				0.32
<3 times a month	Ref			
1–2 times a week	0.62	0.10	3.65	0.60
3–6 times a week	1.95	0.30	12.60	0.49
Once a day	1.53	0.21	11.39	0.68
>1 time a day	0.53	0.05	5.23	0.59
**Fatty foods**				0.86
<1 time a day	Ref			
Once a day	1.41	0.37	5.45	0.62
>1 time a day	1.09	0.30	3.93	0.90
**Health status in the last month**				
Sick	Ref			
Not sick	5.90	1.18	29.51	0.03 *
**School**				0.27
School 1	Ref			
School 2	0.49	0.18	1.33	0.16
School 3	0.38	0.14	1.04	0.06
School 4	0.47	0.14	1.60	0.23
**Nutritional status**				0.02 *
Normal	Ref			
Severe thinness	1.15	0.20	6.47	0.88
Thinness	0.27	0.06	1.16	0.08
Overweight	0.23	0.05	1.07	0.06
Obese	0.01	0.00	0.22	0.04
**Physical activity**				0.22
Very light	Ref			
Light	1.86	0.89	3.89	0.10
Moderate	0.57	0.12	2.64	0.47
Vigorous	2.46	0.38	16.03	0.35
Body image	0.84	0.67	1.06	0.14
Influence of friends	0.94	0.88	1.00	0.06
Influence of parents	1.01	0.95	1.06	0.82
**Component Theory of Planned Behavior**				
Attitude	1.92	0.52	7.15	0.33
Subjective norms	1.25	0.37	4.04	0.71
Control behavior	0.89	0.32	2.50	0.83
Intention	0.87	0.40	1.87	0.71
**Adolescents’ eating habits**				
**Carbohydrates**				
Once a day	Ref			
>1 time a day	1.33	0.26	6.74	0.73
**Vegetables**				0.49
<1 time a day	Ref			
Once a day	1.88	0.65	5.43	0.24
>1 time a day	1.37	0.44	4.20	0.59
**Fruits**				0.98
<1 time a day	Ref			
Once a day	1.15	0.28	4.65	0.85
>1 time a day	1.13	0.09	14.55	0.93
**Animal-based protein sources**				0.00 *
<1 time a day	Ref			
Once a day	0.60	0.22	1.62	0.32
>1 time a day	4.47	1.47	13.57	0.01
**Plant-based protein sources**				0.59
<3 times a month	Ref			
1–2 times a week	0.48	0.02	10.57	0.64
3–6 times a week	1.15	0.06	21.73	0.93
Once a day	1.36	0.07	27.17	0.84
>1 time a day	1.46	0.06	34.37	0.82
**Sweet snacks**				0.55
<3 times a month	Ref			
1–2 times a week	0.34	0.02	5.82	0.46
3–6 times a week	0.56	0.04	8.88	0.68
Once a day	0.51	0.03	9.29	0.65
>1 time a day	0.93	0.06	15.66	0.96
**Sweet beverages**				0.64
<3 times a month	Ref			
1–2 times a week	3.43	0.65	18.25	0.15
3–6 times a week	2.65	0.53	13.33	0.24
Once a day	3.358	0.68	16.62	0.14
>1 time a day	2.95	0.46	18.81	0.25
**Salty foods**				0.21
<3 times a month	Ref			
1–2 times a week	14.18	1.38	146.23	0.03
3–6 times a week	7.60	0.80	72.16	0.08
Once a day	7.86	0.77	80.24	0.08
>1 time a day	4.74	0.45	50.05	0.20
**Fatty foods**				0.89
<3 times a week	Ref			
3–6 times a week	1.33	0.07	24.75	0.85
Once a day	2.00	0.12	32.49	0.63
>1 time a day	1.55	0.09	26.38	0.76

## Data Availability

The datasets generated during and/or analyzed during the current study are available from the corresponding author on reasonable request.

## References

[1] Patton G.C., Olsson C.A., Skirbekk V., Saffery R., Wlodek M.E., Azzopardi P.S., Stonawski M., Rasmussen B., Spry E., Francis K. (2018). Adolescence and the next generation. Nature.

[2] Lassi Z., Moin A., Bhutta Z. (2017). Nutrition in Middle Childhood and Adolescence. Disease Control Priorities, (Volume 8): Child and Adolescent Health and Development.

[3] Soliman A.T., Alaaraj N., Hamed N., Alyafei F., Ahmed S., Shaat M., Itani M., Elalaily R., Soliman N. (2022). Review Nutritional interventions during adolescence and their possible effects. Acta Biomed..

[4] Trübswasser U., Talsma E.F., Ekubay S., Poelman M.P., Holdsworth M., Feskens E.J.M., Baye K. (2022). Factors Influencing Adolescents’ Dietary Behaviors in the School and Home Environment in Addis Ababa, Ethiopia. Front. Public Health.

[5] Chacón V., Liu Q., Park Y., Rohloff P., Barnoya J. (2021). Diet quality, school attendance, and body weight status in adolescent girls in rural Guatemala. Ann. N. Y. Acad. Sci..

[6] Keats E.C., Rappaport A., Jain R., Oh C., Shah S., Bhutta Z.A. (2018). Diet and Eating Practices among Adolescent Girls in Low-and Middle-Income Countries A Systematic Review. Nutrients.

[7] Health Research and Development Unit (2018). Indonesia Basic Health Research.

[8] Health Research and Development Unit (2013). Indonesia Basic Health Research.

[9] Sawyer A.D.M., van Lenthe F., Kamphuis C.B.M., Terragni L., Roos G., Poelman M.P., Nicolaou M., Waterlander W., Djojosoeparto S.K., Scheidmeir M. (2021). Dynamics of the complex food environment underlying dietary intake in low-income groups: A systems map of associations extracted from a systematic umbrella literature review. Int. J. Behav. Nutr. Phys. Act..

[10] FAO, IFAD, UNICEF, WHO (2020). The State of Food Security and Nutrition in The World: Transforming Food Systems For Affordable Healthy Diets.

[11] Mukanu M.M., Delobelle P., Thow A.M., Mchiza Z.J.-R. (2022). Determinants of dietary patterns in school going adolescents in Urban Zambia. Front. Nutr..

[12] Noll M., de Mendonça C.R., de Souza Rosa L.P., Silveira E.A. (2017). Determinants of eating patterns and nutrient intake among adolescent athletes: A systematic review. Nutr. J..

[13] Joulaei H., Keshani P., Kaveh M.H. (2018). Nutrition literacy as a determinant for diet quality amongst young adolescents: A cross sectional study. J. Nutr. Intern. Megazine.

[14] Global Nutrition Cluster Tips on Nutrition Interventions for the Humanitarian Response Plan 2016. https://reliefweb.int/report/world/tips-nutrition-interventions-humanitarian-response-plan-june-2016..

[15] FAO, IFAD, WFP (2015). The State of Food Insecurity in the World 2015: Meeting the 2015 International Hunger Targets: Taking Stock of Uneven Progress.

[16] Cochran W.G. (1977). Sampling Techniques.

[17] Roosita K. (2015). Pengembangan Indeks Gizi Seimbang Untuk Menilai Kualitas Konsumsi Pangan Remaja Usia 13–18 Tahun Di Indonesia (Development of Balance Diet Indices to Assess Quality of Food Consumption in Indonesian Adolescents Aged 13–18 Years Old). Master Thesis.

[18] Individual Food Consumption Survey Team (2014). Food Picture.

[19] Ministry of National Education Republic of Indonesia (2003). Decree No. 20-2003, National Education System.

[20] BKKBN (1998). Gerakan Keluarga Berencana dan Keluarga Sejahtera.

[21] Sharma R. (2013). The Family and Family Structure Classification Redefined for the Current Times. J. Fam. Med. Prim. Care.

[22] Wang W.C., Worsley A. (2014). Healthy eating norms and food consumption. Eur. J. Clin. Nutr..

[23] Kemenkes R.I. (2014). Pedoman Gizi Seimbang.

[24] Fadare O., Mavrotas G., Akerele D., Oyeyemi M. (2019). Micronutrient-rich food consumption, intra-household food allocation and child stunting in rural Nigeria. Public Health Nutr..

[25] Coates J., Swindale A., Bilinsky P. (2007). Household Food Insecurity Access Scale (HFIAS) for Measurement of Food Access: Indicator Guide.

[26] Ashari C.R. (2017). Studi Analisis Ketahanan Pangan pada Rumah Tangga Miskin Perkotaan dan Perdesaan di Sulawesi Selatan. Ph.D. Thesis.

[27] Sallis J.F., Grossman R.M., Pinski R.B., Patterson T.L., Nader P.R. (1987). The development of scales to measure social support for diet and exercise behaviors. Prev. Med..

[28] Makiabadi E., Asadollahi A., Kaveh M.H., Salehi M. (2019). Psychometric Properties of Persian Version of Nutrition Literacy Inventory (NLI-28, 2017) among University Students. Int. J. Nutr. Sci..

[29] Dewi N.U., Khomsan A., Dwiriani C.M., Riyadi H., Ekayanti I., Nurulfuadi N. (2022). Validity and reliability of the theory of planned behavior questionnaire on the balanced dietary behavior of adolescents in a post-disaster area. J. Health Sci..

[30] Thompson M.A., Gray J.J. (1995). Development and Validation of a New Body-Image Assessment Scale. J. Pers. Assess..

[31] Ministry of Health of Republic of Indonesia (2020). Decree No. 2-2020, Standards for Children Anthropometry.

[32] FAO, WHO, UNU (2001). Human Energy Requirements: Report of a Joint FAO/WHO/UNU Expert Consultation.

[33] Goldberg G.R., Black A.E., Jebb S.A., Cole T.J., Murgatroyd P.R., Coward W.A., Prentice A.M. (1991). Critical evaluation of energy intake data using fundamental principles of energy physiology: 1. Derivation of cut-off limits to identify under-recording. Eur. J. Clin. Nutr..

[34] Hoteit M., Mohsen H., Yazbeck N., Diab S., Sarkis J., Sacre Y., Hanna-Wakim L., Bookari K. (2022). Household Food Insecurity, Anemia, Malnutrition and Unfavorable Dietary Diversity among Adolescents: Quadruple Whammies in the Era of Escalating Crises in Lebanon. Nutrients.

[35] Yannakoulia M., Lykou A., Kastorini C.M., Saranti Papasaranti E., Petralias A., Veloudaki A., Linos A. (2016). Socio-economic and lifestyle parameters associated with diet quality of children and adolescents using classification and regression tree analysis: The DIATROFI study. Public Health Nutr..

[36] Işık Balcı Y., Karabulut A., Gürses D., Ethem Çövüt I. (2012). Prevalence and Risk Factors of Anemia among Adolescents in Denizli, Turkey. Iran. J. Pediatr..

[37] UNICEF (2019). UNICEF Global Databases: Infant and Young Child Feeding.

[38] Adesogan A.T., Havelaar A.H., McKune S.L., Eilittä M., Dahl G.E. (2020). Animal source foods: Sustainability problem or malnutrition and sustainability solution? Perspective matters. Glob. Food Sec..

[39] Downs S.M., Fraser S.N., Storey K.E., Forbes L.E., Spence J.C., Plotnikoff R.C., Raine K.D., Hanning R.M., McCargar L.J. (2012). Geography Influences Dietary Intake, Physical Activity and Weight Status of Adolescents. J. Nutr. Metab..

[40] Man C.S., Salleh R., Ahmad M.H., Baharudin A., Koon P.B., Aris T. (2020). Dietary Patterns and Associated Factors Among Adolescents in Malaysia: Findings from Adolescent Nutrition Survey 2017. Int. J. Environ. Res. Public Health.

[41] Ronca D.B., Blume C.A., Cureau F.V., Camey S.A., Leotti V.B., Drehmer M., Schaan B.D., de Carvalho K.M.B. (2020). Diet quality index for Brazilian adolescents: The ERICA study. Eur. J. Nutr..

[42] Agustina R., Rianda D., Setiawan E.A. (2021). Relationships of Child-, Parents-, and Environment-Associated Determinants with Diet Quality, Physical Activity, and Smoking Habits Among Indonesian Urban Adolescents. Food Nutr. Bull..

[43] Tay J.E.F., Kaur S., Tham W.W., Gan W.Y., Ya N.N.C., Tan C.H., Tung S.E.H. (2022). Food security and diet quality among urban poor adolescents in Kuala Lumpur, Malaysia. Nutr. Res. Pract..

[44] Vanhelst J., Béghin L., Duhamel A., De Henauw S., Ruiz J.R., Kafatos A., Androutsos O., Widhalm K., Mauro B., Sjöström M. (2017). Do adolescents accurately evaluate their diet quality? The HELENA study. Clin. Nutr..

[45] De Miguel-Etayo P., Moreno L.A., Santabárbara J., Martín-Matillas M., Azcona-San Julian M.C., Marti Del Moral A., Campoy C., Marcos A., Garagorri J.M. (2019). Diet quality index as a predictor of treatment efficacy in overweight and obese adolescents: The EVASYON study. Clin. Nutr..

[46] Raynor H.A., Goff M.R., Poole S.A., Chen G. (2015). Eating Frequency, Food Intake, and Weight: A Systematic Review of Human and Animal Experimental Studies. Front. Nutr..

[47] Flieh S.M., Miguel-Berges M.L., Huybrechts I., Breidenassel C., Grammatikaki E., Donne C.L., Manios Y., Widhalm K., Molnár D., Stehle P. (2023). Food portion sizes and their relationship with energy, and nutrient intakes in adolescents: The HELENA study. Nutrition.

[48] Christofaro D.G.D., Tebar W.R., Mota J., Fernandes R.A., Scarabottolo C.C., Saraiva B.T.C., Delfino L.D., de Andrade S.M. (2020). Gender Analyses of Brazilian Parental Eating and Activity With Their Adolescents’ Eating Habits. J. Nutr. Educ. Behav..

[49] Liu K.S.N., Chen J.Y., Ng M.Y.C., Yeung M.H.Y., Bedford L.E., Lam C.L.K. (2021). How Does the Family Influence Adolescent Eating Habits in Terms of Knowledge, Attitudes and Practices? A Global Systematic Review of Qualitative Studies. Nutrients.

[50] Bargiota A., Delizona M., Tsitouras A., Koukoulis G.N. (2013). Eating habits and factors affecting food choice of adolescents living in rural areas. Hormones.

[51] Groele B., Głąbska D., Gutkowska K., Guzek D. (2019). Mothers’ Vegetable Consumption Behaviors and Preferences as Factors Limiting the Possibility of Increasing Vegetable Consumption in Children in a National Sample of Polish and Romanian Respondents. Nutrients.

[52] Kołłątaj W., Sygit K., Sygit M., Karwat I.D., Kołłątaj B. (2011). Eating habits of children and adolescents from rural regions depending on gender, education, and economic status of parents. Ann. Agric. Environ. Med..

[53] Reardon T., Boughton D., Tschirley D., Haggblade S., Dolislager M., Minten B., Hernandez R. (2015). Urbanization, Diet Change, and Transformation of the Downstream and Midstream of the Agrifood System: Effects on the Poor in Africa and Asia. Faith Econ..

[54] Ragelienė T., Grønhøj A. (2020). The influence of peers′ and siblings′ on children’s and adolescents′ healthy eating behavior. A systematic literature review. Appetite.

[55] Shao Y., Kang S. (2022). The association between peer relationship and learning engagement among adolescents: The chain mediating roles of self-efficacy and academic resilience. Front. Psychol..

[56] van der Velde L.A., van Dijk W.W., Numans M.E., Kiefte-de Jong J.C. (2022). Extending the Theory of Planned Behavior for Explaining Dietary Quality: The Role of Financial Scarcity and Food Insecurity Status. J. Nutr. Educ. Behav..

[57] Vasiljevic M., Ng Y.-L., Griffin S.J., Sutton S., Marteau T.M. (2016). Is the intention–behaviour gap greater amongst the more deprived? A meta-analysis of five studies on physical activity, diet, and medication adherence in smoking cessation. Br. J. Health Psychol..

[58] Rogol A.D., Clark P.A., Roemmich J.N. (2000). Growth and pubertal development in children and adolescents: Effects of diet and physical activity. Am. J. Clin. Nutr..

[59] Sokolowski C.M., Higgins S., Vishwanathan M., Evans E.M. (2020). The relationship between animal and plant protein intake and overall diet quality in young adults. Clin. Nutr..

[60] Scaglioni S., De Cosmi V., Ciappolino V., Parazzini F., Brambilla P., Agostoni C. (2018). Factors Influencing Children’s Eating Behaviours. Nutrients.

[61] Pfledderer C.D., Gren L.H., Metos J., Brusseau T.A., O’Toole K., Buys S.S., Daly M.B., Frost C.J. (2021). Mothers’ Diet and Family Income Predict Daughters’ Healthy Eating. Prev. Chronic Dis..

[62] Nutbeam D. (2000). Health literacy as a public health goal: A challenge for contemporary health education and communication strategies into the 21st century. Health Promot. Int..

[63] Miller L.M.S., Cassady D.L. (2015). The effects of nutrition knowledge on food label use. A review of the literature. Appetite.

[64] Scalvedi M.L., Gennaro L., Saba A., Rossi L. (2021). Relationship Between Nutrition Knowledge and Dietary Intake: An Assessment Among a Sample of Italian Adults. Front. Nutr..

[65] Pan L., Mu M., Yang P., Sun Y., Wang R., Yan J., Li P., Hu B., Wang J., Hu C. (2020). Clinical Characteristics of COVID-19 Patients with Digestive Symptoms in Hubei, China: A Descriptive, Cross-Sectional, Multicenter Study. Am. J. Gastroenterol..

[66] Keller H., Allard J., Vesnaver E., Laporte M., Gramlich L., Bernier P., Davidson B., Duerksen D., Jeejeebhoy K., Payette H. (2015). Barriers to food intake in acute care hospitals: A report of the Canadian Malnutrition Task Force. J. Hum. Nutr. Diet..

[67] Kedir S., Hassen K., Melaku Y., Jemal M. (2022). Determinants of overweight and/or obesity among school adolescents in Butajira Town, Southern Ethiopia. A case-control study. PLoS ONE.

[68] Liberali R., Kupek E., Assis M.A.A. (2019). de Dietary Patterns and Childhood Obesity Risk: A Systematic Review. Child. Obes..

